# The Salop Infirmary, Shrewsbury

**Published:** 1904-11-19

**Authors:** 


### THE SALOP INFIRMARY, SHREWSBURY.

#### GREAT IMPROVEMENT IN BUSINESS MANAGEMENT AND FINANCES.

We congratulate the Committee and all interested in this institution upon the new form of annual report which they have just issued. This report shows clearly the position of the hospital, the sources from which its subscriptions come, the actual subscribers to the infirmary, the number of subscribers resident in, and the number of patients sent from each parish, so that the managers are now in a position to make an organised effort to increase the funds of the infirmary by showing definite results based upon provable facts.

It will be remembered that this institution fell into serious financial straits a few years ago. Sir Henry Burdett was consequently called in to advise the trustees and directors and to prepare a new scheme which would place the hospital in a sound financial position. Sir Henry Burdett's recommendations were ultimately adopted, with the result that the Committee are able to report for the year ended June 30th, 1904, that instead of a deficiency the accounts show a balance of £1,381. The directors express special satisfaction on the substantial growth that has taken place in the item of annual subscriptions, which shows an increase of £691 for the year. The expenditure upon house expenses has been reduced by upwards of £180, and the directors have been able to repay upwards of £500 of the £2,200 withdrawn from capital last year. The "Uniform System of Accounts" has been adopted as recommended by Sir Henry Burdett, and the directors are able to report :—"The experience of the first year's working of the new regulations leads the directors to believe that, while a permanent increase of income has accrued to the infirmary, its sphere of usefulness has not been thereby in the smallest degree diminished."

The report, which is excellently printed and turned out, does not contain a table of contents, nor a list of the names and addresses of all the trustees or governors of the hospital. No doubt these omissions will be supplied next year.

We congratulate the directors and governors and all concerned upon the changes and improvements which have been introduced at Shrewsbury without the slightest friction of any kind, though they have placed the hospital in a state of much greater efficiency, have introduced business methods into the management, and have secured for the institution greater prosperity than it has ever enjoyed before. There are a number of similar county and other hospitals where changes identical to those made at the Salop Infirmary could not fail to improve the financial and general management. It is, therefore, the more remarkable that so much stagnation should still exist in connection with several county and extra-metropolitan hospitals of importance to the community.

